# Pulsatile Tinnitus as a Manifestation of Radiation-Induced Internal Carotid Artery Stenosis in a Nasopharyngeal Carcinoma Survivor: A Case Report

**DOI:** 10.7759/cureus.84762

**Published:** 2025-05-24

**Authors:** Yun-Chiang Chen, Shiang-Ru Lu, Yi-Chi Wang, Shu-Yu Yang, Pei-Chi Hsiao

**Affiliations:** 1 Department of Physical Medicine and Rehabilitation, Chi Mei Medical Center, Tainan, TWN; 2 Department of Neurology, Kaohsiung Medical University Hospital, Kaohsiung, TWN; 3 Department of Medical Imaging, Chi Mei Medical Center, Tainan, TWN

**Keywords:** carotid stenosis, carotid stent, nasopharyngeal carcinoma, radiation, tinnitus

## Abstract

Nasopharyngeal carcinoma (NPC) is prevalent in East and Southeast Asia, with radiotherapy being the primary treatment modality. Carotid artery stenosis is a serious but often overlooked complication of radiotherapy. We present the case of a middle-aged NPC survivor who developed progressive symptoms, including left facial numbness, dysphagia, tongue atrophy, postural dizziness, and pulsatile tinnitus, 18 years after concurrent chemoradiation. Carotid ultrasound revealed high-grade stenosis of the left cervical internal carotid artery. Following carotid stenting, his dizziness and tinnitus resolved. This case illustrates the insidious progression of carotid stenosis after radiotherapy and underscores the importance of early vascular assessment in post-radiation patients with atypical symptoms such as pulsatile tinnitus. Clinicians should maintain a high index of suspicion in at-risk individuals, and physical therapists should adopt precautionary rehabilitation strategies to minimize the risk of vascular complications.

## Introduction

Nasopharyngeal carcinoma (NPC) is a malignancy originating from the mucosal lining of the nasopharynx. Radiation therapy (RT) remains the cornerstone of management, and advances in RT technology greatly reduce radiation-related toxicities. As survival outcomes improve, managing late radiation complications becomes increasingly vital to high-quality care in NPC survivors.

Among these complications, carotid vasculopathy is particularly concerning. Radiation can compromise vascular integrity through various mechanisms, ultimately leading to carotid artery stenosis and cerebrovascular events [1]. Recent data indicate that NPC survivors face an elevated risk of stroke, with onset occurring about a decade earlier than in the general population [2].

Carotid stenosis often remains clinically silent until significant cerebral hypoperfusion develops. Typical symptoms include transient monocular visual loss (amaurosis fugax) or hemispheric manifestations (i.e., unilateral motor or sensory disturbances and speech dysfunction). Atypical symptoms like dizziness, diplopia, amnesia, and headache are often under-recognized, hindering early detection [3].

This report describes a case of NPC in which various post-radiation symptoms emerged years after treatment, with pulsatile tinnitus leading to the diagnosis of severe internal carotid artery (ICA) stenosis. It underscores the need for early recognition and management of vascular complications in this population.

## Case presentation

A 37-year-old Taiwanese man with well-managed hypertension, but no history of diabetes mellitus, dyslipidemia, obesity, smoking, alcohol use, or betel nut chewing, was diagnosed with stage III NPC, initially staged as T3N2cM0. He underwent concurrent chemoradiation from March to September 1998 and received regular follow-ups and treatment at our otorhinolaryngology, oral surgery, and rehabilitation outpatient clinics. Since January 2006, left facial numbness had been noted during follow-up.

He developed tinnitus in his left ear in September 2015, followed by dysphagia and neck tightness in May 2016. A videofluoroscopic swallow study conducted in May 2016 revealed a well-functioning oral phase but an impaired pharyngeal phase, characterized by poor hyoid/pharynx movement, weak pharyngeal wall contraction, inadequate tongue base retraction, poor laryngeal closure, and no cricopharyngeal opening. His Penetration-Aspiration Scale score was 8, indicating a high risk of aspiration. At the rehabilitation clinic, massage and manual therapy were applied for neck symptoms, while swallowing training was provided for dysphagia. Despite rehabilitation for dysphagia, he developed aspiration pneumonia in July 2016, necessitating a percutaneous endoscopic gastrostomy in the same month.

**Figure 1:**
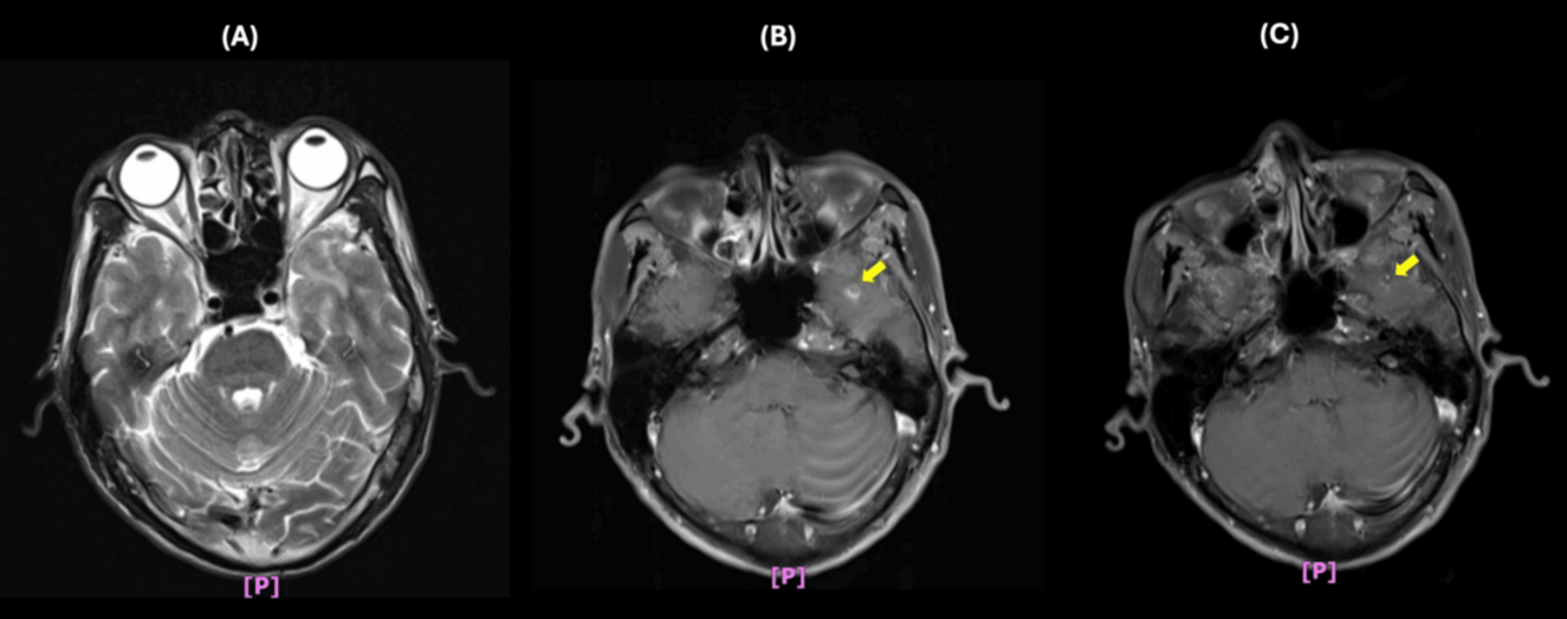
MRI of the nasopharynx (A) May 2017 MRI showing mild white matter changes on T2-weighted imaging. (B) May 2017 MRI revealing an enhancing nodule on the T1 turbo spin echo fat-suppressed sequence (arrow). (C) August 2017 follow-up MRI demonstrating regression of the enhancing nodule. MRI: magnetic resonance imaging

By April 2017, he developed xerostomia, thickened saliva, swollen and oozing gums, and worsening dysphagia, making swallowing saliva difficult. Additionally, he experienced mandible and tongue base numbness. Magnetic resonance imaging (MRI) of the nasopharynx in May 2017 showed mild white matter changes in the left temporal lobe on T2-weighted imaging, along with an enhancing nodule at the left temporal base on the T1 turbo spin echo fat-suppressed sequence (Figure 1A, 1B). A follow-up MRI in August 2017 revealed regression of the enhancing nodule (Figure 1C), supporting a diagnosis of post-radiation effects rather than NPC recurrence. In 2018, he developed tongue muscle atrophy in January and orthostatic hypotension in July, without associated dyspnea, chest pain, or syncope. Collectively, the symptoms that emerged between 2017 and 2018 were initially attributed to delayed RT effects.

**Figure 2:**
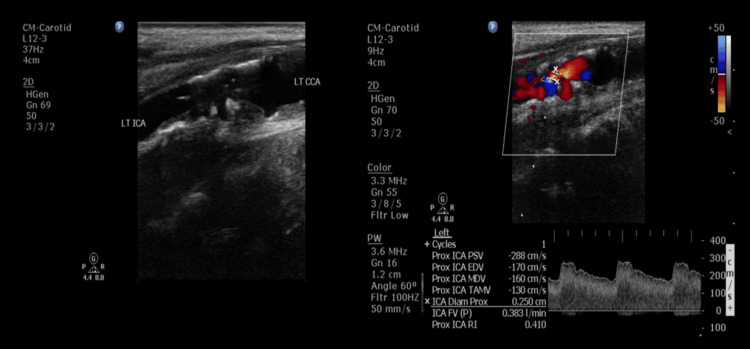
Carotid ultrasound May 2019 carotid sonography revealing atheromatous plaques with elevated mean flow velocities in the left proximal cervical ICA. ICA: internal carotid artery

**Figure 3:**
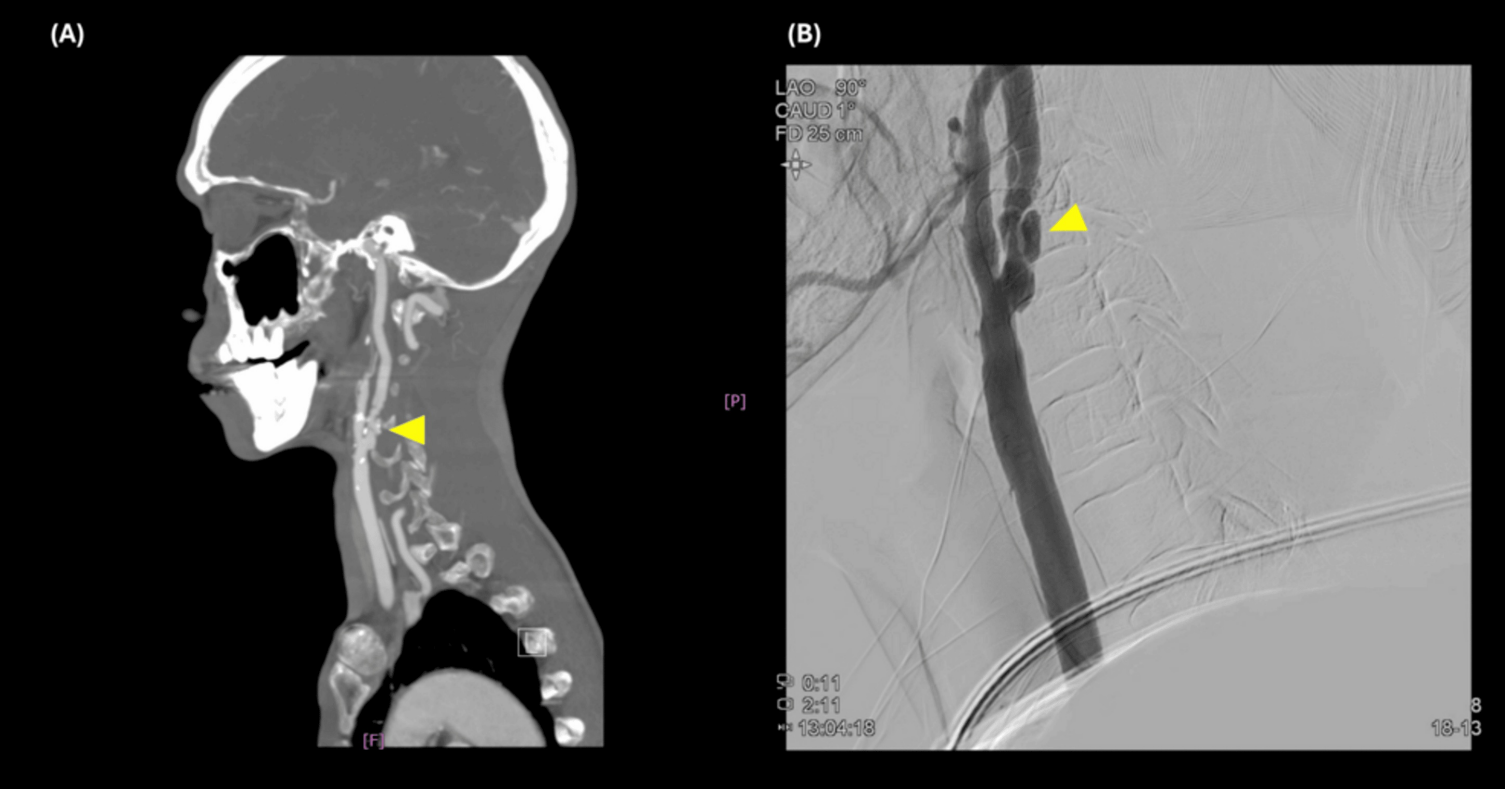
CTA (A) and DSA (B) showing high-grade stenosis (arrowhead) of the left proximal cervical ICA CTA: computed tomography angiography; DSA: digital subtraction angiography; ICA: internal carotid artery

In March 2019, he developed pulsatile tinnitus in the left ear and postural dizziness. These new symptoms prompted his otorhinolaryngologist to order a series of examinations in March 2019. Blood tests showed no evidence of anemia or thyroid dysfunction. Pure tone audiometry revealed bilateral conductive hearing loss, along with high-frequency sensorineural hearing loss in the right ear. Speech audiometry demonstrated good speech discrimination. Auditory brainstem response testing showed bilateral prolongation of waves III and V. Echocardiogram revealed mitral valve prolapse with mild mitral regurgitation. Carotid ultrasound disclosed atherosclerotic changes in the bilateral common carotid arteries, carotid bifurcations (CBs), and ICAs, with high-grade stenosis observed in the left proximal cervical ICA (Figure 2); the vertebral artery flow was adequate, and the ophthalmic artery flow direction remained forward. Computed tomography angiography (CTA) identified atherosclerosis with ulcerative plaque and significant segmental narrowing (>80%) in the left proximal cervical ICA (Figure 3A), as well as mild segmental narrowing (<50%) in the right proximal cervical ICA and CB.

**Figure 4:**
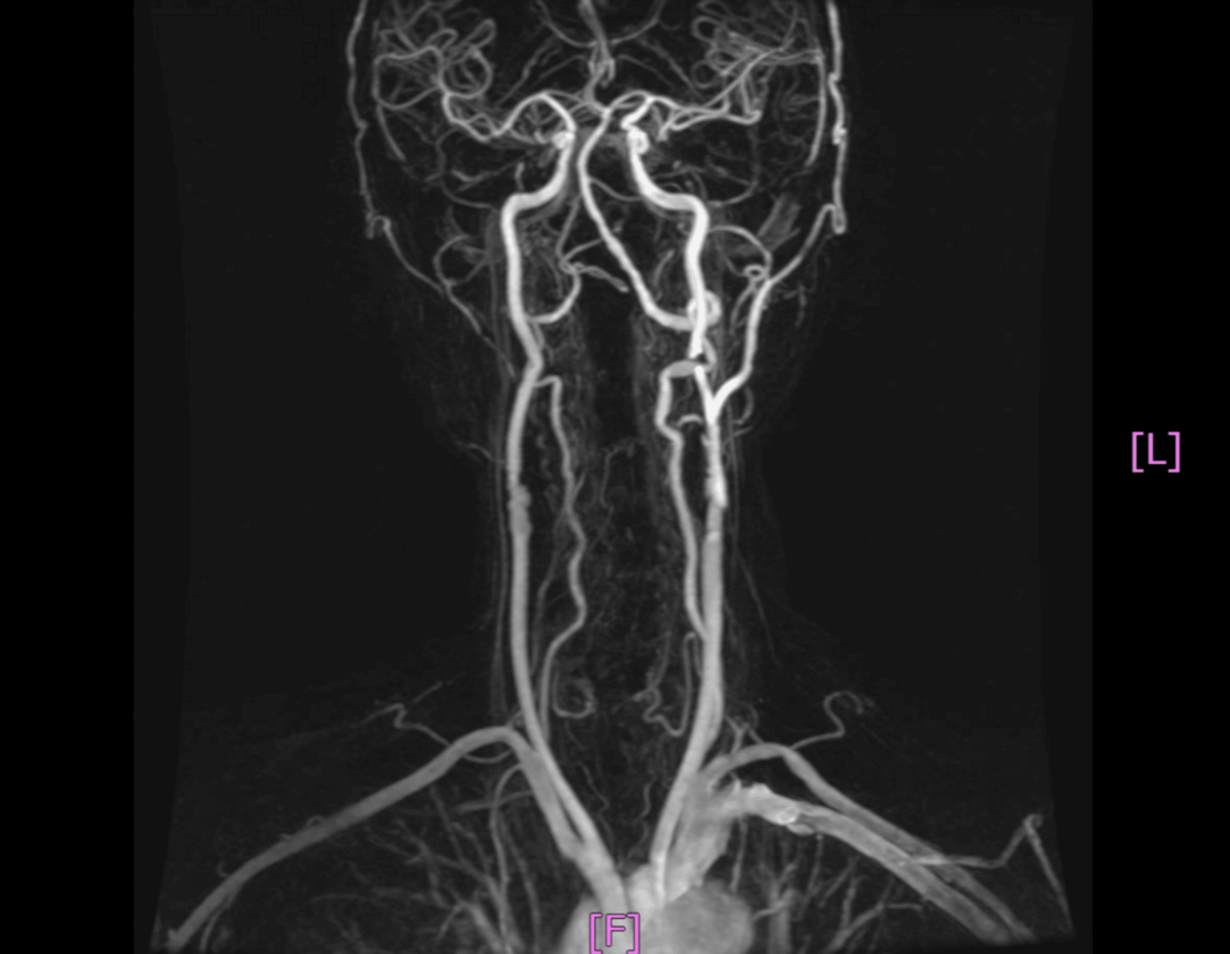
Neck MRA follow-up Neck MRA in July 2020 confirming sustained patency of the left ICA following post-stenting. MRA: magnetic resonance angiography; ICA: internal carotid artery

Given the diagnosis of high-grade stenosis of the left ICA, he was admitted in May 2019 for evaluation of carotid stenting. Digital subtraction angiography (DSA) confirmed 90% eccentric stenosis of the left proximal ICA (Figure 3B). Dual antiplatelet therapy was initiated, followed by a percutaneous transluminal carotid stenting with a Wallstent. He tolerated the procedure without complications, and his hemodynamic status remained stable. Following carotid stenting, his dizziness and pulsatile tinnitus resolved immediately. During subsequent outpatient visits, he reported no recurrence of these symptoms; however, his dysphagia and other complaints persisted. Antiplatelet and antihypertensive therapies were continued. Neck MR angiography (MRA) in July 2020 demonstrated maintained patency of the left ICA following stent placement (Figure 4). 

**Figure 5:**
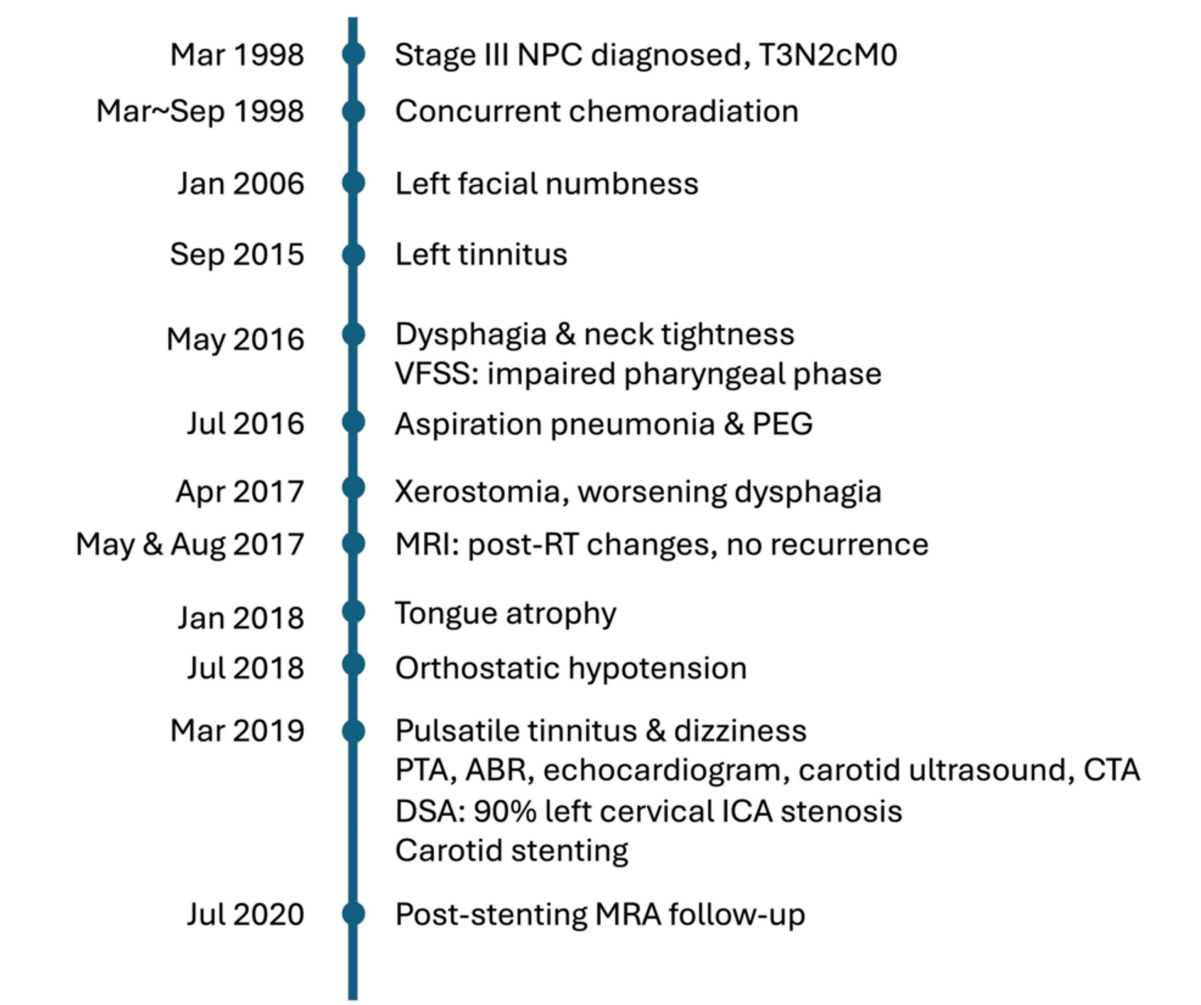
Case report timeline, made in accordance with CARE guideline NPC: nasopharyngeal carcinoma; VFSS: videofluoroscopic swallow study; PEG: percutaneous endoscopic gastrostomy; MRI: magnetic resonance image; RT: radiation therapy; PTA: pure tone audiometry; ABR: auditory brainstem response; CTA: computed tomography angiography; DSA: digital subtraction angiography; ICA: internal carotid artery; MRA: magnetic resonance angiography; CARE: CAse REports

The timeline of his clinical evolution with diagnostic tests and treatments is shown in Figure 5.

## Discussion

This case illustrates the broad spectrum of late radiation complications in NPC survivors. Xerostomia, gum swelling, and neck tightness likely arose from radiation damage to the mucosa, soft tissues, and muscles. Facial and tongue base numbness may reflect radiation-induced trigeminal and glossopharyngeal neuropathies or neck-tongue syndrome [4]. Dysphagia appeared multifactorial, possibly due to post-radiation fibrosis and lower cranial nerve injury. Postural dizziness and orthostatic hypotension raised concerns for autonomic dysfunction [5], as RT can impair baroreceptor function essential for blood pressure regulation [6]. While dizziness and tinnitus are common in NPC survivors [7], pulsatile tinnitus is rare and warrants careful evaluation for potential underlying pathology. 

Pulsatile tinnitus can arise from systemic conditions (i.e., anemia, hyperthyroidism, valvular heart disease, or intracranial hypertension), local non-vascular causes (such as tumors or bony dysplasia), or local vascular abnormalities (including arterial stenosis, dissection, aneurysms, fibromuscular dysplasia, aberrant arteries, and dural arteriovenous fistula) [8]. In our patient, systemic and local non-vascular causes were largely ruled out through clinical evaluation, making a local vascular etiology the most probable explanation. Subsequent evaluation confirmed significant carotid artery stenosis, and the prompt resolution of pulsatile tinnitus following carotid stenting strongly supported a vascular etiology as the underlying cause. Stenosis of the ICA can create turbulent blood flow, generating vibrations in the vessel wall. Given the close proximity of the ICA to the cochlea, these vibrations can be transmitted to the inner ear and perceived as sound [9]. Typically, stenosis linked to pulsatile tinnitus occurs in the petrous portion of the ICA but can also be found in the extracranial segment [10], as illustrated in this case.

Carotid artery stenosis is a common yet often under-recognized complication of RT in NPC survivors. A meta-analysis of 16 studies reported a 22.8% incidence of significant (>50%) carotid stenosis in post-RT NPC patients, compared to 5.6% in control subjects [11]. In our patient, hypertension was well-controlled, and no other cardiovascular risk factors were present, making RT the most likely explanation for his premature carotid narrowing. For early detection, carotid ultrasound is a reliable screening tool, while DSA remains the gold standard for evaluating vascular stenosis [1]. Based on recent recommendations, annual carotid ultrasound screening should begin one year after RT for head and neck cancer patients [12]. 

Management of post-RT carotid artery stenosis generally follows standard guidelines for non-irradiated patients, focusing on lifestyle modifications and risk factor control. Antiplatelet agents reduce stroke risk, while statins help slow stenosis progression and lower stroke rates [13]. In symptomatic severe cases, carotid endarterectomy or stenting may be considered. Endarterectomy carries higher risks of cranial nerve injury and wound infection [1], whereas stenting is prone to thrombosis and restenosis [14].

Oncologic rehabilitation plays a crucial role in managing the late effects of RT, particularly dysphagia and musculoskeletal dysfunction. Several compensation strategies, such as effortful swallow, Mendelsohn maneuver, and shaker exercise, are often used to improve swallowing function. Cervical manipulation and deep tissue massage are often employed to relieve musculoskeletal pain, improve function, and enhance quality of life. These maneuvers are generally safe but can carry certain risks in patients with severe ICA stenosis. For instance, head positioning maneuvers (neck extension/flexion), a technique to facilitate swallowing, might compromise cerebral blood flow [15]. Manual techniques around the anterior neck might dislodge carotid plaques, leading to embolic strokes in patients with carotid stenosis [16,17]. A study on chronic neck pain patients found that maximal cervical rotation and manipulation reduced vertebral artery blood flow, though cerebral hemodynamics remained unchanged [18]. Nonetheless, computational fluid-solid coupling models indicate that rotational manipulation significantly alters blood flow dynamics in the Circle of Willis if ICA stenosis exceeds 90% [19]. 

Given the risks of carotid artery stenosis in post-RT NPC patients, physical therapists should work closely with clinicians to identify at-risk individuals, particularly those who show symptoms of postural dizziness, pulsatile tinnitus, or unexplained neurological symptoms. It is advisable to avoid aggressive cervical manipulation and instead use alternative, safer rehabilitation strategies, such as gentle myofascial release, postural correction, and targeted swallowing therapy. 

## Conclusions

Pulsatile tinnitus may serve as a warning sign of severe radiation-induced carotid artery stenosis in NPC survivors. Otolaryngologists play a key role in identifying such vascular complications. Regular carotid ultrasound screening is recommended for early detection, with timely intervention reducing the risk of stroke. Physiatrists and physical therapists should tailor rehabilitation plans to ensure the safety of these patients.
